# A Case of Aneurism of the Crural Artery

**Published:** 1795

**Authors:** Thompson Forster

**Affiliations:** Surgeon on the Staff of the Army, and Surgeon to Guy's Hospital.


### VII. *A Cafe of Aneurifm of the Crural Artery; communicated in a Letter to Dr.* Simmons, *by Mr.* Thompfon Forfter, *Surgeon on the Staff of the Army, and Surgeon to Guy's Hofpital.*

TO DR. SIMMONS.

Dear Sir,

TO the two cafes of Aneurifm which you have done me the honour to infert in the fifth volume of Medical Facts and Obfervations, I am defirous of adding the following, as I flatter myfelf it will tend ftill further to elucidate the peculiar utility and advantages of the operation in queftion.

Believe me, Dear Sir,

Your's, &c.

*Nov.* 3, 1794. THOMPSON FORSTER.

CASE.

### CASE.

Lawrence M'Carthy, a labouring man, aged thirty-feven years, was admitted, as my patient, into Guy's Hofpital, on the 30th of July 1794, for the cure of an aneurifm of the crural artery.

About nine months before his admiffion, he had perceived a fmall tumor on his right thigh, near that part where the crural artery dips under the triceps mufcle; as it occafioned no inconvenience, nor prevented his working, he took but little notice of it; it came fpontaneoufly, without any external violence, and remained ftationary for near fix months before it became painful: when the tumor had acquired the fize of an egg, a pulfation was perceptible in it, but not before.

At this period of the difeafe he was advifed to foment the part, and to make ufe of liniments: this he continued to do for fome time; but finding no relief from thefe remedies, he applied to a furgeon, who recommended the ufe of a bandage, which he made ufe of for near three months, but without any abatement of the pain; and the tumor in the mean time had increafed to a very confiderable

ſize, and the limb in general had acquired ſomething more than its natural bulk.

The patient, naturally hypochondriacal, became anxious, irritable, and dejected; complaining of great pain in the limb, and particularly in the tumor, which was in ſome meaſure eaſed by preſſure. In this ſtate he came into the hoſpital; and his general habit having been lowered by bleeding, purgatives, and a ſuitable regimen previouſly to the operation, I performed it on Monday, the 11th of Auguſt, by making an inciſion in the courſe of the lower edge of the ſartorius muſcle, and about an inch below where the profunda is uſually given off. Having laid bare the artery, * I paſſed a ligature under it with an eyed probe, and applying the ſtick, ſurrounded by adheſive plaſter, &c. as deſcribed in the former caſes †, the ar-

* With a view of conveying to the reader a more preciſe idea of the operation, I have made a ſketch of the parts concerned in it, from a ſubject diſſected for the purpoſe. See the annexed engraving (plate 1, fig. 2.) in which *a* refers to Poupart's ligament; *b* to the crural artery, with a ligature paſſed under it at the part where it was tied; *c* to the profunda; and *d* to the ſartorius muſcle. It ſeems hardly neceſſary to remind the reader that the object of this ſketch being merely to point out the ſeat of the operation, the parts are delineated in their natural ſtate.

† Vide Vol. V. p. 6.

tery tery was thus ſurrounded, and by theſe means equally compreſſed; the pulſation below of courſe ceaſed: but, for fear of a ſudden hæmorrhage, I paſſed a ſecond ligature about half an inch above the former, laying it looſe, that an aſſiſtant might inſtantly tie it in caſe of ſuch an accident.

Auguſt 21ſt. The firſt ligature, with the ſtick, came away with eaſe.

Auguſt 22d. The ſecond ligature came away with equal eaſe.

An account of the ſtate of the pulſe at the wriſt, and of the temperature of both limbs, at the ham, and at the foot, was taken every day with great accuracy by Mr. G. Babington, according to the annexed Table *, until Auguſt the 27th, when the temperature of each was found to be equal.

The ſize of the tumor gradually decreaſed, and the patient, having the perfect uſe of his limb, was diſmiſſed, cured, October 10, 1794.

The preceding caſe differs materially from the two former, not only in the circumſtance of the tumor in this having been ſituated in the

* See page 119.

upper part of the thigh, ſo that the artery could not be ſecured lower than about an inch below where the profunda is uſually given off, but likewiſe in the very great pain the patient endured both night and day for three weeks before the operation. The tumor was as conſiderable, but the enlargement of the limb below it was much leſs than in the former caſes. After the operation, the ſymptoms were much ſlighter than in the other caſes, probably owing to the low ſtate I thought it proper to reduce the patient to for the purpoſe; and the ligature came away on the tenth day after the operation without the leaſt trouble. But the circumſtance in which it differed the moſt eſſentially from the other two, was, that the tumor was completely abſorbed in ſeven weeks, and the patient had then acquired a perfect uſe of the limb, while, in the former caſes, the patients did indeed acquire the uſe of their limbs, but the tumors, though leſſened and free from pulſation, ſtill remained.

TABLE.

### TABLE.

| Day of the Month. | pulse at wrist. | tem. of arm. | tem. right ham. | right foot. | left ham. | left foot. | Time of day when the obs. were made. |
|---|---|---|---|---|---|---|---|
| August 11 | | 68½° | 98° | 94 ° | 97 ° | 96 ° | 10½ P. M. |
| 12 | | 68½ | 97 | 91 | 91 | 89 | 8½ A. M. |
| | 128 | 70 | 99 | 91 | 94 | 93 | 10½ P. M. |
| 13 | 109 | 68 | 98 | 92 | 90 | 88 | 8½ A. M. |
| | 112 | 71 | 100 | 95 | 98 | 95 | 10½ P. M. |
| 14 | 104 | 68 | 98 | 91 | 91 | 91½ | 8½ A. M. |
| | 116 | 72 | 99 | 96 | 96 | 96 | 8¼ P. M. |
| 15 | 96 | 69 | 97 | 91 | 94 | 88 | 8¼ A. M. |
| | 112 | 72½ | 97 | 93 | 94 | 95 | 8 P. M. |
| 16 | 97 | 72 | 98 | 93½ | 94 | 90 | 8 A. M. |
| | 112 | 73 | 98 | 95 | 94 | 94 | 8 P. M. |
| 17 | 96 | 71 | 98 | 92 | 95 | 89 | 8 A. M. |
| | 112 | 74 | 97 | 94 | 94 | 94 | 8 P. M. |
| 18 | 92 | 70 | 93 | 91 | 92½ | 89 | 8 A. M. |
| | 110 | 72 | 97 | 92 | 94 | 93 | 9 P. M. |
| 19 | 100 | 68 | 94 | 90 | 92 | 91 | 8 A. M. |
| | 124 | 71½ | 101 | 96½ | 97 | 97 | 8½ P. M. |
| 20 | 114 | 67 | 100 | 93 | 96 | 94 | 8½ A. M. |
| | 116 | 70 | 99 | 95 | 95 | 94 | 8½ P. M. |
| 21 | First ligature and stick came away with ease, there being a perfect solution of continuity. | | | | | | |
| | 100 | 66 ° | 97° | 8° | 93 ° | 86 ° | 8 A. M. |
| | 100 | 69 | 98 | 92 | 95 | 94 | 8 P. M. |
| 22 | Second ligature was removed. | | | | | | |
| | 100 | 69° | 96° | 86 ° | 93 ° | 84 ° | 8½ A. M. |
| | 108 | 69 | 98 | 94 | 97 | 95 | 9 P. M. |
| 23 | 100 | 67½ | 96 | 91 | 93 | 90 | 9 A. M. |
| | 104 | 70½ | 98 | 94 | 95 | 93 | 8½ P. M. |
| 24 | 96 | 69 | 97 | 89 | 95 | 87 | 10 A. M. |
| | 104 | 69 | 99 | 95 | 98 | 95 | 8 P. M. |
| 25 | 104 | 66½ | 98 | 93 | 96 | 92 | 8½ A. M. |
| | 106 | 69½ | 95 | 92 | 93 | 91 | 8 P. M. |
| 26 | 100 | 64 | 96 | 90 | 91 | 86 | 8 A. M. |
| | 106 | 66 | 98 | 92 | 94 | 90 | 8½ P. M. |
| 27 | 100 | 64 | 96 | 91 | 92 | 91 | 8½ A. M. |
| | 95 | 63½ | 96 | 90 | 96 | 90 | 8 P. M. |

